# Associations Between Pet Ownership and Attitudes Toward Pets With Youth Socioemotional Outcomes

**DOI:** 10.3389/fpsyg.2018.02304

**Published:** 2018-11-26

**Authors:** Kristen C. Jacobson, Laura Chang

**Affiliations:** Department of Psychiatry and Behavioral Neuroscience, University of Chicago, Chicago, IL, United States

**Keywords:** pets, children, depressed mood, delinquency, empathy, prosocial behavior

## Abstract

Evidence regarding the effects of pet ownership and related variables on youth socioemotional development is mixed. Inconsistencies across studies may be due to a variety of factors, including the use of different outcomes measured across studies, small potential effect sizes, and use of selected samples. In addition, studies have not systematically controlled for demographic characteristics that may bias results, nor have studies systematically examined whether effects are consistent across different subgroups. The present study examined the impact of pet ownership and attitudes toward pets on four measures of youth socioemotional outcomes: delinquency, depressed mood, empathy, and prosocial behavior. Linear mixed-effect regression analyses were conducted on 342 youth (48.0% male) aged 9–19 (*M* = 14.05, *SD* = 1.77) from a racially, ethnically, and socioeconomically diverse sample. The majority (59.1%) of youth currently lived with a dog or cat and all participants completed the Pet Attitude Scale-Modified. Pet owners reported lower delinquency and higher empathy than non-owners; however, group differences became non-significant once demographic factors were controlled for. Attitudes toward pets was significantly associated with all four outcomes. More positive attitudes was modestly associated with lower delinquency (β = -0.22, *p* < 0.001) and higher empathy (β = 0.31, *p* < 0.001), with smaller effects for depressed mood (β = -0.12, *p* = 0.04) and prosocial behavior (β = 0.12, *p* = 0.02). For delinquency, empathy, and prosocial behavior, effects were only slightly attenuated and remained statistically significant after controlling for gender, age, race/ethnicity, family socioeconomic status, and pet ownership, although the effect for depressed mood became non-significant after inclusion of these demographic factors. While there was some variability in effect sizes across different subgroups, none of the interactions between attitudes toward pets and gender, race/ethnicity, age, family SES, or pet ownership was statistically significant, indicating that the effects may transcend individual differences in demographic characteristics. Overall, the study adds to a growing body of work supporting a positive relationship between emotional bonds with pets and youth socioemotional outcomes and offers potential explanations for inconsistencies across previous studies.

## Introduction

There is a high prevalence of pet ownership in the United States, with dogs and cats being the most common types of pets (American Pet Products Association [APPA], 2018). Overall, roughly 68% of United States household have pets (American Pet Products Association [APPA], 2018), and pet ownership is even more common among families with children (Melson, 2003; Westgarth et al., 2007, 2010), According to the US Census (2017), approximately 57% of households with children have two or more children. Thus, it is likely that more American children live with pets than with siblings. There is also evidence that humans form strong emotional bonds with pets. In a recent large United States national survey of adults, more than 80% of cat and dog owners indicated that “companionship, love, company, affection” was a positive benefit of owning a pet (American Pet Products Association [APPA], 2018). Studies of both children and adults reveal that a significant number of individuals consider pets to be family members (Melson, 2001; Cohen, 2002; American Pet Products Association [APPA], 2018) and to rank relationships with pets as being important (Kosonen, 1996). A study of 7- to 8-year-old children reported that pets ranked higher as sources of social support than non-immediate family members such as aunts, uncles and grandparents, (McNicholas and Collis, 2000), while a study of 12-year olds found that children reported greater satisfaction with their relationships with pets than with their relationships with siblings (Cassels et al., 2017). Despite the prevalence and importance of pets in children’s lives, there is surprisingly little research on the effects of pets on child development, especially in comparison to research examining human–human family relationships.

While there have been numerous reviews on the impact of therapy animals on child developmental outcomes (e.g., Nimer and Lundahl, 2007; Lentini and Knox, 2009; O’Haire, 2013; Chur-Hansen et al., 2014), only one published review has considered the effects of pets. This comprehensive evidence review reported only 22 studies of child pet ownership and related pet variables (e.g., time spent with pets, attachment toward pets) published between 1960 and 2016 (Purewal et al., 2017). According to this review, evidence for positive benefits of pets is inconsistent across studies. Of the 39 results summarized from these 22 studies, 64% (*N* = 25) claimed positive effects, although more than 25% of these results (*N* = 7) did not include associated *p*-values or confidence intervals. Exactly one-third (*N* = 13) reported no differences between owners and non-owners, and one result showed a negative impact of pet ownership.

One possible explanation for inconsistencies in prior research is that studies have used different measures of child developmental outcomes. Pet ownership and related variables are most often studied in relationship to child self-esteem and measures of social competence. While the majority of studies examining self-esteem have reported positive results, studies of other measures of social competence have been less consistent (Purewal et al., 2017). In a large study of 826 Croatian children aged 10–15, greater attachment to pet dogs was associated with higher empathy and more prosocial behavior (Vidović, 1999). Two studies of Canadian elementary children also found that dog ownership was associated with greater empathy, but empathy levels were actually lower among cat-owners (Daly and Morton, 2003, 2006). Pet ownership has been associated with lower self-reported loneliness in two unique samples: a study of 293 racially and ethnically diverse, rural, high school students living in Arizona (Black, 2012) and a study of 332 homeless youth living in Los Angeles (Rhoades et al., 2015), although there were no effects of pet ownership or attachment to pets on perceived loneliness in the large Croatian study (Vidović, 1999).

Research on pet ownership and child emotional and behavioral problems is less common, and results are considerably more mixed. Vidović (1999) found no relationship between pet ownership and anxiety in a large sample of Croatian youth. Gadomski et al. (2015) found that rural children aged 4–10 currently living with pets had lower screening anxiety scores than non-pet-owning children, but did not find a relationship between pet ownership with broader measures of parent-reported youth emotional, behavioral, and attentional problems. Rhoades et al. (2015) reported that pet-owning homeless youth reported less depression than non-pet-owning youth, but a large study of Australian adolescents did not find an association between pet ownership and a composite measure of child emotional, social, and school problems (Mathers et al., 2010). One of the only longitudinal studies of pet ownership found that levels of tearfulness in 8- to 12-year olds were decreased at 12 months following adoption of a pet dog, in comparison to non-dog owning children, although the sample size for this study was small (Paul and Serpell, 1996). There are virtually no published studies of pet ownership and child behavior problems, although there are multiple reports suggesting that child hyperactive, aggressive, and disruptive behaviors in school decrease after introduction of pets into classrooms (e.g., Hergovich et al., 2002; Kotrschal and Ortbauer, 2003; Tissen et al., 2007; O’Haire et al., 2013). In the longitudinal study, Paul and Serpell (1996) reported a decrease in “naughty” behavior among children at 1 month following the adoption of the family dog, but this effect did not persist at the 6- or 12-month assessments. Given the relatively limited number of studies on pet ownership in childhood, it is unclear whether inconsistencies across studies are due to the use of different outcome measures or due to differences in sample characteristics. Research designs that include multiple measures of child socioemotional outcomes within the same sample are an efficient way to test whether positive benefits of pet ownership are limited to certain outcomes.

Prior studies also differ markedly in whether or not they control for demographic covariates. Given that pet ownership is not randomly distributed across families, it is critically important that studies consider other factors that might account for results. For example, ownership of and interest in pets tend to peak in middle childhood (i.e., 8–12 years) and to decline during adolescence (Melson, 1988; Paul and Serpell, 1992, 1996). Because rates of depression and delinquency increase during adolescence, correlations between pet ownership and outcomes could be driven by these coinciding developmental patterns, especially when samples encompass a wide age range. Gender confounds are also under-explored. Less than half of the studies reported in the Purewal et al. (2017) review controlled for gender, despite there being marked gender differences in behavioral, social, and emotional problems in childhood and adolescence.

Race/ethnicity and socioeconomic factors are other important factors to consider. Within North America, Caucasian families are more likely to have companion animals than African American, Hispanic, and Asian families (Siegel, 1995; Risley-Curtiss et al., 2006; Pet Food Industry, 2012; Saunders et al., 2017). While there is evidence that dogs are equally valued among Hispanic and Caucasian adolescents (Black, 2012) and adults (Johnson and Meadows, 2002; Schoenfeld-Tacher et al., 2010), Caucasian adults tend to own more pets and be more highly attached to their pets than African Americans (Brown, 2003). Racial/ethnic differences have not been routinely examined in studies of children, however, as most studies have contained more than 95% Caucasian youth. While population-based studies in Europe typically report inverse associations between family pet ownership and levels of income and education (Mullersdorf et al., 2010; Murray et al., 2010; Westgarth et al., 2010), a study of over 42,000 adults living in California reported that several positive socioeconomic factors, such as full-time employment, higher income, and home ownership, predicted both dog and cat ownership (Saunders et al., 2017). Because cultural and economic factors are important predictors of both pet ownership and child outcomes, failure to control for these effects could lead to biased results.

Finally, we have limited information as to whether the effects of pets on child development vary for children in different subgroups. One reason for this gap is that prior research on the impact of pets in children has relied heavily on small sample sizes that lack diversity. Of the 22 studies reported in Purewal et al. (2017) review, 40.9% (*N* = 9) were based on sample sizes less than 100 individuals, with 5 of these using less than 25 participants. In particular, the vast majority of prior work has been based on Caucasian samples. Thus, whether the potential protective effects of pets generalize to minority youth is largely unknown. There is also evidence that emotional bonds with pets may vary by gender (Kidd and Kidd, 1989; Johnson et al., 1992; Woodward and Bauer, 2007), age (Melson, 1988; Paul and Serpell, 1992, 1996), and family composition (Melson et al., 1991; Siegel, 1995; Bodsworth and Coleman, 2001; Westgarth et al., 2013). Thus, it is important to test whether the benefits of pet ownership vary across demographic characteristics.

The present study was designed to address these limitations. Specifically, detailed measures of pet ownership and attitudes toward pets were added to a larger study of risk and protective factors for youth socioemotional and behavioral outcomes in a racially, ethnically, and socioeconomically diverse sample of urban and suburban youth aged 8–19. We obtained multiple measures of child socioemotional outcomes and caregivers of youth provided detailed information on demographic characteristics. This enabled us to address the following research questions: (1) Is there a stronger relationship with youth socioemotional outcomes for attitudes toward pets compared to pet ownership? (2) Do the effects of pet ownership and attitudes toward pets generalize across different socioemotional outcomes? (3) Are effects attenuated when demographic confounds are considered? (4) Are the effects different for youth in various ecological niches?

## Materials and Methods

### Participants and Procedure

Participants in this study took part in an in-lab family study at the University of Chicago. The sample was recruited from a larger community-based study of 3,582 urban and suburban youth in the greater Chicago area who had participated in a prior in-school survey of socioemotional behavior among middle school students (Chen and Jacobson, 2013). The in-lab study consisted of 378 youth aged 8–19 from 241 families, including 137 sibling pairs. More than 85% of families contacted for recruitment agreed to participate in the in-lab assessment, which occurred between March 2010 and August 2012. Exclusion criteria included the presence of severe physical, psychological, or neurological problems in children which would have interfered with study participation (<2% of families contacted) and/or a primary caregiver who could not read or write English (∼6% of families contacted). The study protocol was approved by the University of Chicago Institutional Review Board. In accordance with the Declaration of Helsinki, a parent/legal guardian (79.4% biological mothers) provided written informed consent for themselves and their children and youth provided written informed assent. Participants were compensated for their time. Youth and a single caregiver were studied simultaneously in an on-campus research laboratory during a single 3–4-h visit. Assessments included face-to-face interviews with caregivers and self-report instruments administered to both youth and caregivers.

### Measures

#### Predictors

##### Pet ownership

Pet ownership was assessed through a detailed, semi-structured interview with the youth’s caregiver that was designed for the current study. In brief, caregivers were asked to report on the presence of any pets currently living in the home, as well as any other pets they had had during the past 10 years. Questions were asked about dogs, cats, and small pets, including mammals, reptiles, birds, and fish. Preliminary analyses (available from first author) indicated that youth who lived only with small pets were more similar demographically to youth who did not live with any pets than they were to youth living with a dog or a cat. Likewise, youth living with a cat were similar to youth living with a dog. Thus, analyses used current dog and/or cat ownership as the primary predictor. Pet ownership information was available for 371 out of 378 youth.

##### Attitudes toward pets

Youth completed the Pet Attitude Scale-Modified (PAS-M; Templer et al., 1981). This measure includes 18 questions and assesses participants’ general attitudes about pets. Responses ranged from 1 = strongly disagree to 7 = strongly agree. Questions were phrased both positively (e.g., “You should treat your house pets with as much respect as you would a human member of your family”) and negatively (e.g., “The world would be a better place if people would stop spending so much time caring for their pets and stated caring more for other human beings instead”). Negatively phrased questions were reverse-coded, and all items were averaged to create a single composite score (Cronbach’s α = 0.90), with higher scores indicating more positive attitudes toward pets. Youth were given the PAS-M scale regardless of whether or not they were current or past pet owners. Due to a procedural error, the PAS-M was not administered to *N* = 28 out of 378 youth (7.4%). Youth with more than 20% missing data on individual items were given a missing value for the composite score.

##### Demographic factors

Youth age was calculated using caregiver reports of youth date of birth subtracted from the date of the study day. Gender and race/ethnicity were obtained via both youth and caregiver report. For race/ethnicity, youth and their caregivers were asked whether they were Hispanic or Non-Hispanic, and they used a checklist to indicate their racial background. Responses included White, Black or African American, Asian, Native Hawaiian or Other Pacific Islander, American Indian or Alaskan Native, and more than one race. Family socioeconomic status (SES) was assessed with the two-factor Hollingshead weighted SES index based on parental education and occupation, with a possible range of 8–66. The SES measure correlated positively with caregiver report of family income (*r* = 0.62, *N* = 373, *p* < 0.001) and was used because it was less negatively skewed (skewness = -0.56) than income levels (skewness = -2.76).

#### Youth Outcomes

##### Prosocial behavior

Youth prosocial behavior was assessed using the Child Social Behavior Scale (CSBS; Crick, 1996) based on caregiver report on child. The CSBS uses four items to assess child prosocial behavior toward peers (e.g., “This child tries to cheer up peers when they are sad about something”). Responses ranged from 1 = never true to 5 = always true. Items were averaged to create a mean composite score (Cronbach’s α = 0.91) with higher scores indicating more prosocial behavior. Youth with more than 25% missing data on individual items were given a missing value for the composite score.

##### Empathy

Youth empathy was assessed through self-report using the 15-item Social Attitudes Scale (SAS; Eisenberg et al., 1996). Responses ranged from 1 = really like me to 3 = not at all like me. Questions were phrased both positively (e.g., “I feel sorry for other kids who don’t have toys and clothes”) and negatively (e.g., “I think it is funny that some people cry during a sad movie or while reading a sad book”). Positively phrased questions were reverse-coded so that higher scores indicated greater empathy, and all items were averaged to create a composite score (Cronbach’s α = 0.91). Youth with more than 20% missing data on individual items were given a missing value for the composite score.

##### Depressed mood

Youth self-report depressed mood were assessed using the 20-item Center for Epidemiological Studies Depression Scale (CES-D; Radloff, 1977). Questions asked how often each statement was true during the past week and included items such as “you felt depressed,” “you were bothered by things that usually didn’t bother you,” and “you enjoyed life” (reverse-coded). Responses were 1 = never or rarely to 4 = most of the time or all of the time. Items were averaged to create a mean score (Cronbach’s α = 0.87) with higher scores indicating greater depressed mood. Youth with more than 20% missing data on individual items were given a missing value for the composite score.

##### Delinquency

Youth delinquency was measured with 16 items assessing frequency of a broad range of illegal (e.g., stealing something worth more than $50), norm-violating (e.g., skipping school without permission), and aggressive (e.g., getting into a serious physical fight) behaviors within the past 12 months. Responses were given on a 3-point scale, ranging from 0 = never to 3 = five or more times; each behavior was recoded into 0 = never and 1 = one or more times. A composite score of the number of delinquent behaviors endorsed was computed by summing the recoded responses to the 16 items (Cronbach’s α = 0.81). The initial composite delinquency score was positively skewed (skewness = +1.68); thus the composite score (+1) was log-transformed to normality (skewness = -0.13). Youth with more than 20% missing data on individual items were given a missing value for the summary score.

### Statistical Analyses

The data analysis for this paper was generated using SAS software, version 9.3 for Windows. Copyright © 2002–2010, SAS Institute Inc. SAS and all other SAS Institute Inc. product or service names are registered trademarks or trademarks of SAS Institute Inc., Cary, NC, United States. Descriptive statistics and preliminary analysis of demographic differences were calculated using standard chi-square tests, *t*-tests, and Pearson correlations. Primary analyses used hierarchical multiple regression to test the effects of pet ownership and attitudes toward pets on youth outcomes. Because the sample consists of a subsample of sibling pairs, regression analyses were conducted using linear mixed models in SAS PROC MIXED. Mixed level models take into account the clustering of siblings within families by including family ID as a random effect, while all predictors are modeled as fixed effects. All regression models described in the results adjusted for the non-independence of the sample. Separate analyses were conducted for pet ownership versus attitudes toward pets, and separate analyses were conducted for each of the four youth outcomes. Both unstandardized (b) and standardized (β) regression coefficients are reported, as the latter further serves as a measure of effect size, roughly equivalent to Cohen’s *d*.

## Results

### Descriptive Information

#### Missing Data

Of the *N* = 378 youth who participated in the study, 31 youth had missing data on the PAS-M and 5 youth had missing data on pet ownership, resulting in a sample *N* = 342 for statistical analysis. Between 2 and 6 youth were missing data on each outcome, resulting in small differences in sample size across analyses.

#### Demographic Characteristics of Youth

The sample was approximately evenly divided across gender (48.0% male) with a Mean age = 14.05 (*SD* = 1.77; range 9–19). Over half of the sample identified as Hispanic or non-Caucasian, including *N* = 64 Hispanic (18.7%), 121 Black (35.4%), 6 Asian (1.8%), one each American Indian/Alaskan Native and Native Hawaiian/Other Pacific Islander, and 23 youth who reported more than one race (6.7%). Racial/ethnic categories were combined for comparison of minority (63.2%) versus non-Hispanic White (36.8%) youth. The majority (89.8%) of youth lived with their biological mother, and 27.2% lived in single parent homes. There was a wide range of socioeconomic backgrounds (Mean SES = 45.17, *SD* = 13.79, range 9–66).

#### Pet Ownership

Of the 342 youth, 226 (66.1%) currently lived with one or more pets. Dog ownership was most prevalent (*N* = 159, 46.5% of the total sample), followed by cat ownership (*N* = 73, 21.3%) and small pet ownership (*N* = 60, 17.0%). These estimates are largely consistent with figures based on population-based samples (American Pet Products Association [APPA], 2018). For analytic purposes, the sample was divided into current dog and/or cat “owners” (*N* = 202, 59.1%) versus “non-owners,” i.e., youth living with no pets or only small pets (*N* = 140; 40.9%).

Of the 202 owners, 114 lived with dog(s) only, 34 lived with cat(s) only, 20 lived with both dog(s) and cat(s), 15 lived with dog(s) and other small pets, 9 lived with cat(s) and other small pets, and 10 lived with dog(s), cat(s), and other small pets. Of the 140 non-owners, a minority (*N* = 24, 20.7%) currently lived with small pets while the remainder were not currently living with any type of pet. Moreover, almost half of the non-owner group (*N* = 60, 42.9%) had not lived with any type of pet for the past 10 years. Of the *N* = 24 youth living with small pets, the majority (*N* = 19, 79.2%) reported living with fish. Indeed, exactly half (*N* = 12) were living with fish and no other small pets.

### Preliminary Analyses

Chi-square tests and *t*-tests indicated that the *N* = 36 youth excluded due to missing data did not differ significantly from the *N* = 342 included youth on gender, minority racial/ethnic background, age, family SES, pet ownership, or on any of the four youth outcomes (all *p* > 0.10, results available from first author).

Girls (61.2%) were slightly more likely than boys (56.7%) to live with a dog or cat, but the gender difference was not statistically significant (χ^2^ = 0.72, df = 1, *p* = 0.39). Owners and non-owners did not differ in age (*M* = 13.96, *SD* = 1.75 for owners; *M* = 14.18, *SD* = 1.80 for non-owners, *t*_340_ = 1.14, *p* = 0.25). There were significant differences between owners and non-owners in youth racial/ethnic background (χ^2^ = 39.53, df = 1, *p* < 0.001) and family SES (*t*_340_ = 5.09, *p* < 0.001). Minority youth were less likely to own pets than White youth (46.3% versus 81.0%, respectively) and non-owners had lower family SES (*M* = 40.77, *SD* = 14.05) than owners (*M* = 48.22, *SD* = 12.78).

There were no gender difference in self-reported attitudes toward pets (*M* = 5.48, *SD* = 0.91, for females; *M* = 5.45, *SD* = 0.93, for males, *t*_340_ = 0.35, *p* = 0.73). Minority youth reported significantly less positive attitudes toward pets than Caucasian youth (*M* = 5.31, *SD* = 0.95, for minority youth; *M* = 5.73, *SD* = 0.80, for Caucasians, *t*_340_ = 4.12, *p* < 0.001). Age and family SES had modest, albeit significant associations with attitudes, with positive attitudes toward pets decreasing with age (*r* = -0.12, *p* = 0.02) and increasing with higher family SES (*r* = 0.12, *p* = 0.12). Finally, owners reported significantly more positive attitudes toward pets than non-owners (*M* = 5.69, *SD* = 0.83 for owners; *M* = 5.14, *SD* = 0.94 for non-owners, *t*_340_ = 5.66, *p* < 0.001).

**Table 1:** Pearson correlations among study predictors and outcomes.

	Pet ownership	Pet attitudes	Prosocial behavior	Empathy	Depressed mood	Delinquency
Sample *N*	342	342	339	340	336	338
Pet ownership	1.0					
Pet attitudes	0.29***	1.0				
Prosocial behavior	-0.01	0.10#	1.0			
Empathy	0.14**	0.31***	0.15**	1.0		
Depressed mood	-0.08	-0.13*	-0.10#	-0.10#	1.0	
Delinquency	-0.14*	-0.22***	-0.12*	-0.16**	0.38***	1.0
Mean	0.59	5.47	4.30	2.47	1.62	1.04
(SD)	(0.49)	(0.92)	(0.72)	(0.43)	(0.40)	(0.70)

*Pet ownership is a binary variable with 1 = current ownership. Delinquency is shown with log-transformed scores. ^∗∗∗^*p* < 0.001, ^∗∗^*p* < 0.01, ^∗^*p* < 0.05, ^#^*p* < 0.10*.

### Correlations Among Study Outcomes and Predictions

Table 1 presents simple Pearson correlations between main study predictors and outcomes. Note that *p*-values for these correlations are not adjusted for clustered observations of siblings within families. Pet ownership and attitudes toward pets were moderately correlated (*r* = 0.29, *p* < 0.001). There were some significant correlations among the four study outcomes, although most were modest in size, ranging in magnitude from -0.10 to +0.38. Pet ownership was significantly correlated with higher empathy (*r* = 0.14, *p* = 0.008) and lower delinquency (*r* = -0.14, *p* = 0.01). Attitudes toward pets was positively correlated with empathy (*r* = 0.31, *p* < 0.001) and inversely correlated with delinquency (*r* = -0.22, *p* < 0.001) and depression (*r* = -0.13, *p* = 0.02). The correlation between attitudes toward pets and prosocial behavior was smaller (*r* = 0.10) and was significant only at trend level (*p* = 0.08). Overall, correlations between youth outcomes with attitudes toward pets were stronger in magnitude than the respective correlations with pet ownership.

### Pet Ownership and Youth Outcomes

A hierarchical series of mixed level regression models was used to test whether pet ownership was associated with youth socioemotional outcomes. In the first set of models, pet ownership was entered as the sole fixed-level predictor in a simple regression, with family ID entered as a random effect to adjust standard errors and significance tests for the correlated observations. Separate models were run for each outcome. Next, models were re-run including youth gender, age, race/ethnicity, and family SES as covariates.

In the first set of regression models, pet ownership was significantly associated with lower delinquency (*b* = -0.20, *SE* = 0.08, β = -0.29, *t*_121_ = -2.40, *p* = 0.012) and higher empathy (*b* = 0.12, *SE* = 0.05, β = 0.28, *t*_122_ = 2.46, *p* = 0.015). Once demographic covariates were included in the second set of models, effects of pet ownership were not significant for any of the four outcomes.

To determine whether results were influenced by the definition of pet ownership, we ran additional *post hoc* models comparing current dog owners (*N* = 159) with the *N* = 60 youth who had not owned pets in the past 10 years. With demographic factors included in the models, effects using this more extreme definition of pet ownership/non-ownership were not significant for any of the four outcomes.

**Table 2:** Results from regression models of attitudes toward pets predicting youth outcomes.

	Without covariates						With covariates					
	*b*	*SE*	Beta	*t*-value	df	*p*-value	*b*	*SE*	Beta	*t*-value	df	*p*-value
**Prosocial behavior**												
Intercept	3.77	0.23	n/a	16.26	216	<0.001	3.77	0.42	n/a	16.26	216	<0.001
Attitudes	0.10	***0***.***04***	***0***.***12***	***2***.***35***	***121***	***0***.***02***	***0***.***09***	***0***.***04***	***0***.***11***	***2***.***09***	***119***	***0***.***04***
Male							**-*0***.***26***	***0***.***07***	***-0***.***36***	***-3***.***54***	***119***	**<*0***.***001***
Age							-0.01	0.02	-0.03	-0.53	119	0.60
Minority							-0.12	0.10	-0.16	-1.16	119	0.25
SES							-0.001	0.003	-0.01	-0.21	119	0.83
Pet ownership							-0.11	0.10	-0.15	1.17	119	0.25
**Empathy**												
Intercept	1.66	0.14	n/a	12.23	213	<0.001	1.52	0.25	n/a	6.16	214	<0.001
Attitudes	***0***.***15***	***0***.***02***	***0***.***31***	***6***.***02***	***121***	**<*0***.***001***	***0***.***13***	***0***.***02***	***0***.***27***	***5***.***31***	***119***	**<*0***.***001***
Male							***-0***.***20***	***0***.***04***	***-0***.***46***	***-4***.***65***	***119***	**<*0***.***001***
Age							-0.00	0.01	-0.01	-0.25	119	0.80
Minority							***-0***.***12***	***0***.***05***	***-0***.***26***	***-2***.***16***	***119***	***0***.***03***
SES							0.003	0.002	0.09	1.53	119	0.13
Pet ownership							-0.02	0.05	-0.04	0.38	119	0.70
**Depressed mood**												
Intercept	2.52	0.03	n/a	14.21	215	<0.001	1.76	0.25	n/a	7.11	212	<0.001
Attitudes	***-0***.***05***	***0***.***02***	***-0***.***12***	***-2***.***13***	***119***	***0***.***04***	*-*0.04	0.02	*-*0.08	*-*1.49	117	0.14
Male							***-0***.***10***	***0***.***04***	***-0***.***25***	***-2***.***33***	***117***	***0***.***02***
Age							0.01	0.01	0.06	1.05	117	0.29
Minority							0.09	0.05	0.21	1.62	117	0.11
SES							*-*0.003	0.001	*-*0.11	*-*1.76	117	0.08
Pet ownership							*-*0.00	0.05	*-*0.01	0.05	117	0.96
**Delinquency**												
Intercept	1.95	0.23	n/a	8.67	216	<0.001	0.87	0.40	n/a	2.17	213	0.03
Attitudes	***-0***.***17***	***0***.***04***	***-0***.***22***	***-4***.***15***	***120***	**<*0***.***001***	***-0***.***13***	***0***.***04***	***-0***.***17***	***-3***.***21***	***118***	***0***.***002***
Male							0.05	0.07	0.07	0.70	118	0.48
Age							***0***.***09***	***0***.***02***	***0***.***24***	***4***.***85***	***118***	**<*0***.***001***
Minority							*-*0.03	0.09	*-*0.04	0.29	118	0.77
SES							***-0***.***10***	***0***.***003***	***-0***.***20***	***-3***.***18***	***118***	***0***.***002***
Pet ownership							*-*0.04	0.08	*-*0.06	0.51	118	0.61

*Bolded italic values reflect effects significant at *p* < 0.05*.

### Attitudes Toward Pets and Youth Outcomes

Table 2 shows the results from the mixed level regression models used to test whether attitudes toward pets was associated with youth socioemotional outcomes. As above, analyses were run in two steps, without and with demographic covariates. In addition to gender, age, race/ethnicity, and family SES, pet ownership was also included in the second step. Both unstandardized and standardized regression coefficients are shown to enable comparison of estimates within and across models. In models without covariates, attitudes toward pets was significantly associated with all four outcomes. The strongest effects were seen for empathy (β = 0.31, *p* < 0.001) and delinquency (β = -0.22, *p* < 0.001), with more modest effects on prosocial behavior (β = 0.12, *p* = 0.021) and depressed mood (β = -0.12, *p* = 0.035). More positive attitudes toward animals was associated with greater empathy and prosocial behavior and with less delinquency and depressed mood. After including demographic covariates, the association between attitudes toward pets and empathy (β = 0.27), delinquency (β = -0.18) and prosocial behavior (β = 0.11) remained significant at *p* < 0.05, while the effect sizes for depressed mood (β = -0.08) became non-significant (*p* = 0.14). Pet ownership was not significantly associated with any of the four outcomes (all *p* > 0.20).

### Effects of Attitudes Toward Pets Across Different Ecological Niches

We examined whether the relationship between attitudes toward pets and child outcomes differed by gender, racial/ethnic background, or for owners versus non-owners, and whether age or family SES moderated the associations. Moderating effects were tested by including an interaction term between attitudes toward pets with each of the five demographic characteristics. All models were run as mixed level models and controlled for correlations among family members. Each model contained all of the demographic factors (including pet ownership) as main effects, attitudes toward pets as a main effect, and a single interaction term. Models tested each interaction separately, and separate models were run for each outcome. For continuous measures of age and family SES, both attitudes toward pets and the continuous demographic factors were centered prior to creating interaction terms.

Out of the 20 different models (5 interactions × 4 outcomes, results not shown), none of the interaction terms was statistically significant (all *p* ≥ 0.17). To ensure that lack of power to detect statistical interactions did not obscure any meaningful patterns across subgroups, we also ran separate models for each outcome in each subgroup so that we could compare the magnitude of the association between attitudes toward pets and youth outcomes across subgroups. Each regression model included attitudes toward pets and all four of the remaining demographic factors. For example, in addition to attitudes toward pets, the regressions run separately for boys and girls included minority racial/ethnic background, pet ownership, age, and family SES as covariates. For family SES, we used a median split to define subgroups of low versus high SES. For age, separate regressions were run for 9- to 12-year-olds, 13- to 14-year-olds, and 15- to 19-year olds.

**FIGURE 1:**
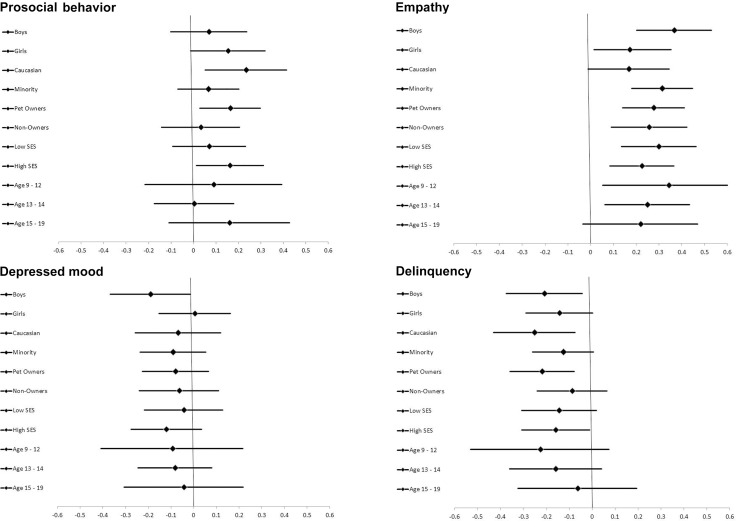
Effect sizes (95% CI) for associations between attitudes toward pets and youth outcomes among different sample subgroups. Error bars reflect 95% confidence intervals around estimates. Effect sizes are based on standardized regression coefficients for attitudes toward pets. Regression models controlled for demographic covariates. Samples sizes vary slightly across outcomes due to missing data: boys (160–164); girls (175–177); Whites (124–126); Non-Whites (211–214); non-owners (137–140); owners (199–200); low SES (164–168); high SES (171–172); age 9–12 (86–89); age 13–14 (148–150); age 15–19 (100–101).

The effect sizes and 95% confidence intervals for the association between attitudes toward pets with child outcomes within each subgroup are presented in Figure 1. While there was some variation in effect sizes across subgroups, differences were modest and there was considerable overlap in confidence intervals.

## Discussion

The current study provided a comprehensive examination of associations between pet ownership and attitudes toward pets with youth socioemotional outcomes. Strengths of the study include: the use of a moderately large, community-based sample of urban and suburban youth with substantial racial, ethnic, and socioeconomic diversity; comparison of results for pet ownership versus youth attitudes toward pets; rigorous measurement of current and history of pet ownership obtained through detailed interviews with caregivers; consideration of multiple socioemotional and behavioral outcomes; the use of sophisticated statistical controlling for potential demographic confounds; and a systematic comparison of effects across different subgroups of youth. Results support three main conclusions: (1) attitudes toward pets is a stronger predictor of youth outcomes than pet ownership; (2) effects are strongest for youth reports of empathy and delinquency compared with prosocial behavior and depressed mood; and (3) significant effects were found among youth across a wide range of demographic characteristics.

### Main Effects

Results from this study add to a small, albeit growing body of work examining the impact of pets on child socioemotional development, and further shed some initial light on potential reasons for inconsistencies across prior studies. First, while there was an initial main effect of pet ownership on child empathy and delinquency, these effects became non-significant once controls for gender, age, minority race/ethnicity, and family socioeconomic status were considered. This underscores the importance of considering demographic confounds in research on pets, given that pet ownership is not randomly distributed across the population. At the same time, controlling for the effects of demographic confounds only slightly attenuated the associations between attitudes toward pets and youth outcomes, and the associations remained statistically significant for three of the four outcomes considered. This result is consistent with both prior theoretical and empirical work suggesting that the positive benefits of pet ownership are largely mediated through the emotional bonds that humans form with animals (Garrity et al., 1989; Friedmann et al., 1993; Sable, 1995; Collis and McNicholas, 1998; Carlisle-Frank and Frank, 2006; Barker et al., 2010; Melson, 2010; Julius et al., 2012; Freund et al., 2016; Purewal et al., 2017). However, it should be noted that the effect sizes for attitudes toward pets were small, ranging in absolute magnitude from β = 0.11 to β = 0.27, after controlling for demographic confounds.

We found the strongest association between attitudes toward pets and child-reported empathy, consistent with a recent empirical review of existing studies on pet ownership and child outcomes (Purewal et al., 2017). In addition, this association was relatively robust across different subgroups of youth in the study. Interestingly, we also found a relatively strong association between attitudes toward pets and youth delinquency. To our knowledge, this may be the first reported significant association between pet-related measures and adolescent externalizing behaviors in a non-clinical sample, although we note that studies examining the impact of introducing pets in classrooms have reported decreases in disruptive behaviors (e.g., Hergovich et al., 2002; Kotrschal and Ortbauer, 2003; Tissen et al., 2007; O’Haire et al., 2013).

We found the smallest effects of attitudes toward pets on prosocial behavior and depressed mood, and the association with depressed mood was not statistically significant once demographic factors were considered. For depressed mood, this result may indicate that relationships with pets only affect certain kinds of emotional problems. For example, a recent study reported that pet ownership was associated with screening anxiety in a sample of rural children, but not with a broader measure of youth socioemotional difficulties (Gadomski et al., 2015). Likewise, Vidović (1999) reported that attachment to pets was associated with child empathy and prosocial behavior in a large sample of Croatian adolescents, but was not significantly associated with anxiety or loneliness. Given the small number of studies that have focused specifically on measures of pediatric anxiety and depression, more work is needed before drawing any firm conclusions about inconsistency of results across different child emotional outcomes. Moreover, we note that the measure of depressed mood used in this study assessed mood experienced during the past week, while the measures of empathy, prosocial behavior, and delinquency encompassed a broader time frame. Rates of depression were also relatively low in our community-based sample. These factors could have reduced potentials associations. Studies that examine pet-related variables in relationship to emotional problems among clinical samples may shed further light on these issues.

For prosocial behavior, the smaller association in comparison to empathy and delinquency was unexpected, given that previous theoretical and empirical work has posited a direct link between attachment to pets, social support, and prosocial behaviors (Collis and McNicholas, 1998; Vidović, 1999; Melson, 2010; Julius et al., 2012; Freund et al., 2016; Purewal et al., 2017). We note that the measure of prosocial behavior we used was based on parent report, and was specific to parent observations of youth prosocial behaviors toward peers, which is a fairly narrow definition of prosocial behavior. Given that this was predominantly a middle- and high-school aged sample, it is possible that child report of a wider range of prosocial behaviors would have been a more appropriate outcome.

### Moderating Effects

This study is one of a handful to systematically explore whether associations between pets and child outcomes were consistent across different demographic subgroups. None of the 20 different interaction terms reached statistical significance (all *p* > 0.15), and there was considerable overlap in the 95% confidence intervals for effect sizes across the different subgroups. This suggests that the positive benefits of pets may transcend individual differences in demographic characteristics. While a larger sample may have revealed statistically significant differences between subgroups, our study demonstrated that differences in effect sizes across subgroups were relatively small, and therefore unlikely to be of meaningful importance.

### Causal Inferences

Although the data from this study are cross-sectional, results may speak to issues of causality. Specifically, there was a significant association between youth self-reports of attitudes toward pets and empathy among youth who did not currently live with a cat or a dog. Indeed, associations between attitudes toward pets and empathy were largely significant across all subgroups examined, even after controlling for demographic factors. Additional *post hoc* analyses (available from first author) indicated that the Pearson correlation between attitudes toward pets and empathy among the 60 youth *who had not lived with any type of pet in the past 10 years* was *r* = 0.27 (*p* = 0.03). Even after age, gender, minority racial/ethnic background, and family SES were included in the model and standard errors adjusted for correlated observations, the effect for attitudes toward pets in this subgroup was significant at a trend level [*F*(1,19) = 3.29, *p* = 0.09]. These results suggest that youth with higher levels of empathy might be more likely to desire pets and to form stronger emotional bonds with pets than youth with lower empathy. This may also be true of other measures of child social and emotional competency, such as self-esteem. On the other hand, we cannot rule out the hypothesis that youth who do not live with pets but who really like pets seek out other opportunities to interact with animals outside the family, which could have a causal effect on empathy.

### Future Research

The fact that the majority of research on the impact of pets on socioemotional and behavioral outcomes in both child and adult samples is based on cross-sectional studies is a significant limitation of the field. While studies that include demographic covariates associated with pet ownership can help control for selection factors, many of the associations between children’s attitudes toward and emotional bonds with pets with children’s social, emotional, and behavioral outcomes are likely to be bi-directional. For example, children who have higher empathy and show more prosocial behavior may be more naturally inclined to form close bonds with pets. Conversely, aggressive children may find it more difficult to form successful relationships with pets, especially if the pet is fearful of the child. Longitudinal studies may help to disentangle the causal nature of these associations, especially if children can be assessed before and after the acquisition of a new pet.

Studies that use within-family designs are also under-utilized in the field of human–animal interactions. Samples that include more than one child per family could shed light on both similarities and differences among children within the same family, and could identify the specific child characteristics that impact the development of emotional bonds with pets. Behavioral genetic designs can further determine the extent to which associations between emotional bonds with pets and outcomes are driven by shared genetic factors. At present, there is only one published study that used a genetically informative design to investigate genetic influence on a pet-related measure. This study found that self-reports of frequency of playing with pets among a middle-aged, male twin sample had a heritability of *h*^2^ = 0.29–0.37, indicating that genetic factors, which are likely mediated through individual differences in personality and related traits, play a role in establishing bonds with pets (Jacobson et al., 2012). Surprisingly, the effects of shared environmental influences, which would include childhood exposure to pets, accounted for less than 10% of the variance in pet play during adulthood. This finding may call into question the causal implications of prior research showing that childhood pet ownership predicts both pet ownership patterns (Serpell, 1981; Westgarth et al., 2010) and strength of emotional bonds with pets in adulthood (Kidd and Kidd, 1989; Ellingsen et al., 2010).

## Limitations and Conclusion

Results from the current study should be considered in the context of several limitations. *First*, attitudes toward pets and outcome measures are based predominantly on youth self-report. Thus, we cannot rule out the hypothesis that factors such as social desirability could account for some of the associations. However, social desirability would not account for the differential patterns of effects seen across outcomes. *Second*, the study focused on general attitudes toward pets, rather than specific emotional bonds with pets. This is because we wanted to directly compare the distal effects of pet ownership with the more proximal, emotional impact of pets, and we needed measures that could be administered to both pet-owning and non-pet-owning youth. The study did obtain measures of emotional bonds with pet dogs from dog-owning youth, and there was substantial overlap between attitudes and emotional bonds among the 154 dog-owning youth who had non-missing data on both measures (*r* = 0.64, *p* < 0.001). Nevertheless, we might have found stronger associations with youth outcomes if we had focused on emotional bonds with specific family pets. *Third*, results may have been confounded by definitions of pet ownership. In particular, sample sizes of youth who lived only with cats and/or small pets were too small to be considered individually. The fact that our findings largely replicated when we used a stricter comparison of current dog owners to youth who had not owned *any pets* in the past 10 years suggest that our results are not biased by our definition; however, larger samples sizes with greater diversity on pet ownership patterns are needed to explore this question more thoroughly. *Fourth*, while our sample contained relatively large numbers of Hispanic and Black youth, sample sizes were too small to determine whether the associations between ownership and attitudes with youth outcomes differed between these two racial/ethnic groups, so youth were combined into a binary variable of minority versus non-minority youth. We have examined racial and ethnic differences in pet ownership and attitudes toward pets in more detail in a separate manuscript, and results suggest that Hispanic and Black youth in this sample show similar patterns, and that both groups show significant differences in comparison to non-Hispanic Caucasian youth (Jacobson and Daly, unpublished). However, we do not know if results would generalize to American youth from other racial and ethnic groups, such as Asian-American or Native American youth. *Finally*, our results may not generalize to other populations. Specifically, pet ownership patterns and demographic correlates of pet ownership vary somewhat between the United States and other countries, so inconsistencies between our results with prior, large-scale studies conducted in other countries could be due to cultural factors. Hispanic youth living in America may also differ from Hispanic youth living in other countries. Cross-cultural studies are needed to disentangle the effects of culture from racial/ethnic background. In addition, our sample is drawn from a predominantly urban and suburban population. Thus, results may not generalize to rural youth. Finally, results are based on a community-based sample. While we did not exclude youth with emotional and behavioral problems from this study, it is possible that the positive benefits of pet ownership, attitude toward pets, and emotional bonds with pets would be greater among patient populations, or among other populations of vulnerable youth.

Despite these limitations, this is one of the only studies to obtain measures of both pet ownership and attitudes toward pets as well as a wide range of socioemotional outcomes in a diverse sample of youth. Our results indicate that pet ownership, *per se*, is unrelated to child outcomes once demographic factors associated with ownership are accounted for. At the same time, controlling for demographic factors had limited impact on the magnitude of associations between attitudes toward pets and child outcomes, and these associations were largely consistent across different subgroups of children. Thus, our study contributes to a growing body of research suggesting that pets may have a positive, albeit modest impact on children.

## Data Availability Statement

The raw data supporting the conclusions of this manuscript will be made available by the authors, without undue reservations, to any qualified researcher.

## Author Contributions

KJ is the principal investigator of the overall project, conceived the study aims and hypotheses, collected and analyzed the data, and wrote the manuscript. LC assisted with literature review.

## Conflict of Interest Statement

The authors declare that the research was conducted in the absence of any commercial or financial relationships that could be construed as a potential conflict of interest.
